# Treatment of dysplastic Barrett’s esophagus using a novel over-the-scope device for hybrid argon plasma coagulation

**DOI:** 10.1055/a-2868-6749

**Published:** 2026-06-01

**Authors:** Francesco Auriemma, Michela Merella, Federica Calabrese, Danilo Paduano, Gianluca Franchellucci, Benedetto Mangiavillano

**Affiliations:** 1 Gastrointestinal Endoscopy UnitHumanitas – Mater DominiCastellanzaItaly; 2 Department of Medicine, Surgery and Pharmacy 9312 University of SassariSassariItaly; 3 Endoscopy UnitIRCCS Humanitas Research HospitalRozzano, MilanItaly

The management of dysplastic Barrett’s esophagus is primarily endoscopic and relies on minimally invasive treatment strategies that combine the resection of visible or nodular lesions with ablation of the remaining flat Barrett’s mucosa 1 .

Endoscopic ablative techniques include radiofrequency ablation (RFA) and alternative methods such as hybrid argon plasma coagulation (APC) and cryotherapy 2 .

Conventional APC is a non-contact thermal ablation technique in which electrical current is delivered through ionized argon gas.

Hybrid APC (H-APC) is a more recent technique that incorporates submucosal fluid injection before ablation, creating a protective cushion that reduces thermal injury to the deeper layers of the esophageal wall. The procedure requires submucosal injection of saline solution, with or without dye, to protect the muscularis propria from thermal damage 3 . Standard APC has been associated with relatively high complication rates, particularly post-treatment strictures. H-APC was introduced to reduce these risks. However, when performed free-hand, maintaining a constant distance from the mucosa can be challenging, potentially resulting in uneven ablation depth.

Among the latest innovations, the ArgoCap system has been developed to improve both the safety and efficacy of H-APC. This device consists of a transparent distal attachment mounted on the tip of the endoscope, designed to stabilize the endoscopic field and maintain a consistent distance from the mucosa during ablation. By ensuring controlled energy delivery, the ArgoCap may reduce the risk of excessive thermal injury and subsequent stricture formation.

**Fig. 1:**
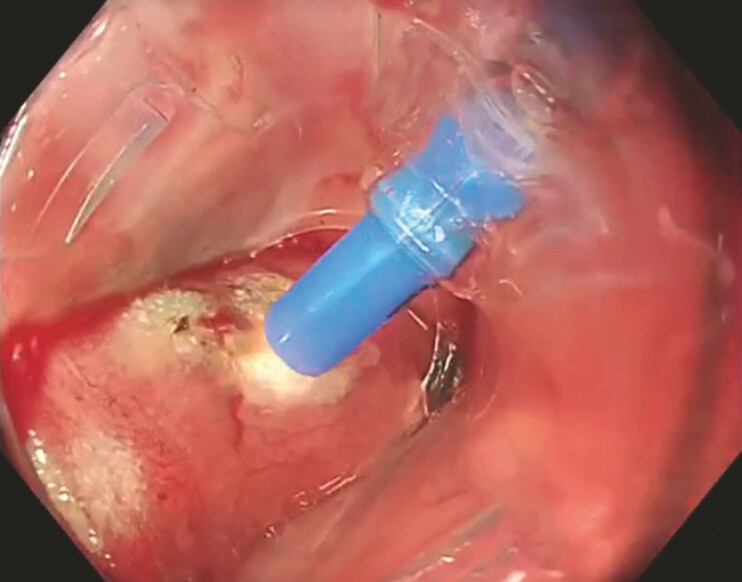
Hybrid APC using the ArgoCap system. APC, argon plasma coagulation.

**Fig. 2:**
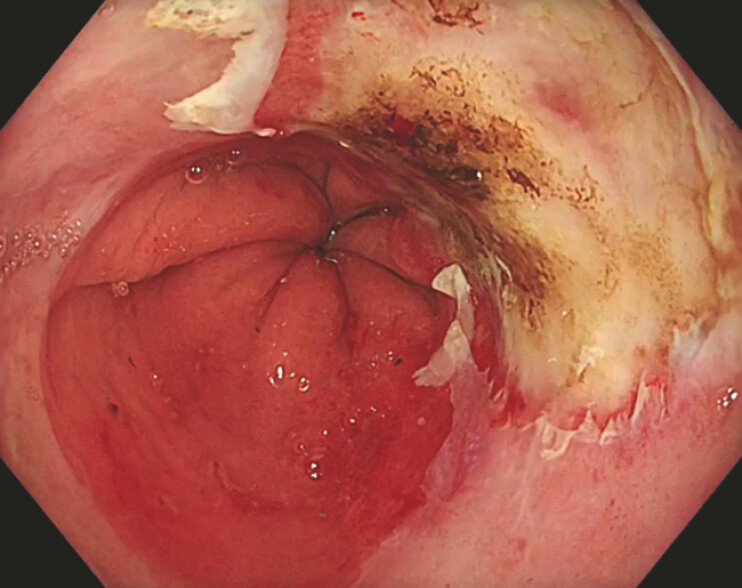
The final result of the ablative treatment.

We report the case of a 76-year-old woman who previously underwent cap-assisted endoscopic mucosal resection for in situ adenocarcinoma arising in dysplastic C3M5 Barrett’s esophagus, followed by RFA. Residual low-grade dysplastic C0M2 Barrett’s esophagus was treated with H-APC using the ArgoCap system ( Video 1 ). After evaluation with acetic acid, diluted methylene blue was injected into the submucosal layer. APC was performed using pulsed mode (effect 4.0, ERBE VIO3), as shown in Fig. 1 and Fig. 2 . The procedure was carried out under deep sedation in an outpatient setting and lasted less than 15 minutes without complications. Follow-up endoscopy showed no evidence of residual intestinal metaplasia.

Treatment of dysplastic Barrett’s esophagus using the ArgoCap system.Video 1

Endoscopy_UCTN_Code_TTT_1AO_2AN
